# Macrophage-specific N-terminal truncation of Plin2 limits the size of lipid droplets

**DOI:** 10.1042/BCJ20253345

**Published:** 2026-01-21

**Authors:** Yaru Tian, Xiaolong Huang, Yuanyuan Wei

**Affiliations:** 1 Department of Immunology, School of Basic Medical Sciences, Fudan University, Shanghai, 200032, China; 2 Shanghai Key Laboratory of Bioactive Small Molecules and State Key Laboratory of Medical Neurobiology, School of Basic Medical Sciences, Fudan University, Shanghai, 200032, China

**Keywords:** lipid droplets, macrophages, Plin2

## Abstract

Lipid droplets (LDs) are dynamic organelles that exhibit cell-type-specific heterogeneity in size, composition, and abundance to support diverse cellular functions. However, the molecular mechanisms regulating this functional diversity remain poorly understood. Here, we identified a cell-type-specific truncated isoform of Plin2, which was present in macrophages but absent in adipocytes. Using N-terminal HA- and C-terminal FLAG-tagged Plin2 constructs combined with immunoprecipitation-mass spectrometry analysis in HEK293T cells or macrophages, we confirmed the N-terminal truncation and mapped the deletion site to residues 40–44. Ectopic expression of this truncated variant significantly reduced LD size in both macrophages and HEK293T cells. These findings reveal that macrophages modulate lipid storage by expressing distinct Plin2 protein variants, suggesting new therapeutic targets for lipid metabolism disorders.

## Introduction

Lipid droplets (LDs), dynamic organelles central to intracellular lipid storage, were initially identified in association with amyloid β in Alzheimer’s disease [1]. These structures have since been observed across diverse organisms, from prokaryotes to multicellular eukaryotes, displaying remarkable variation in size, abundance, and protein composition [2,3]. Structurally, LDs feature a neutral lipid core—predominantly triglycerides and cholesterol esters—encapsulated by a phospholipid monolayer, and their biogenesis begins at specialized endoplasmic reticulum (ER) domains where neutral lipids accumulate. These nascent LDs expand through either de novo lipid synthesis or fusion events. During energy deprivation, LDs undergo degradation via lipolysis or lipophagy [4]. Thus, the formation and turnover of LDs are highly dynamic processes, with LDs themselves exhibiting considerable heterogeneity in size, quantity, and protein/lipid composition across different cell types [5], consequently leading to cell-type-specific functionality of LDs.

Macrophages, key members of the mononuclear phagocyte system, perform essential functions such as phagocytosis, cytokine secretion, and antigen presentation [6,7]. In many infectious and metabolic diseases (e.g., atherosclerosis) as well as cancers, chronic inflammation is often accompanied by macrophage foam cell formation [8]. When macrophages internalize lipids beyond their capacity to maintain intracellular homeostasis, excessive lipid accumulation triggers massive LD formation, giving the cells a characteristic ‘foamy’ appearance. Current LD research mainly examines adipocytes, but due to cell-type LD heterogeneity and functional differences between adipocytes and macrophages, these results cannot be directly applied to macrophages. Therefore, systematic investigations of LDs in macrophages are critical for elucidating the immunological functions of this organelle.

Perilipin (Plin2), a ubiquitously expressed member of the PAT protein family, exhibits nearly exclusive localization to the LD phospholipid monolayer. As the most abundant PAT protein in macrophages [9], aberrant Plin2 expression in macrophages impairs cholesterol efflux, promoting foam cell formation in atherosclerosis [10]. In contrast, *Plin2* knockout mice exhibit resistance to atherosclerosis and diet-induced obesity [11,12]. In macrophages, down-regulation of Plin2 expression promotes the translocation of another PAT protein, Plin3, from the cell membrane to LDs [13], probably because the N-terminus of Plin2 prevents Plin3 from binding to LDs [14]. Moreover, studies have shown that the N-terminal sequence of Plin2 is critical for ubiquitin–proteasome-mediated degradation of cytosolic Plin2 not associated with LDs [14]. However, whether and how Plin2 regulates macrophage-specific LD characteristics remains under investigation.

In the current study, we have discovered a previously unrecognized N-terminal truncated variant of Plin2 in foamy macrophages. Our data suggest that this truncated Plin2 isoform functions to limit LD size in macrophages.

## Results

### Cell-specific truncation of Plin2 occurs in foamy macrophages

**Figure 1:**
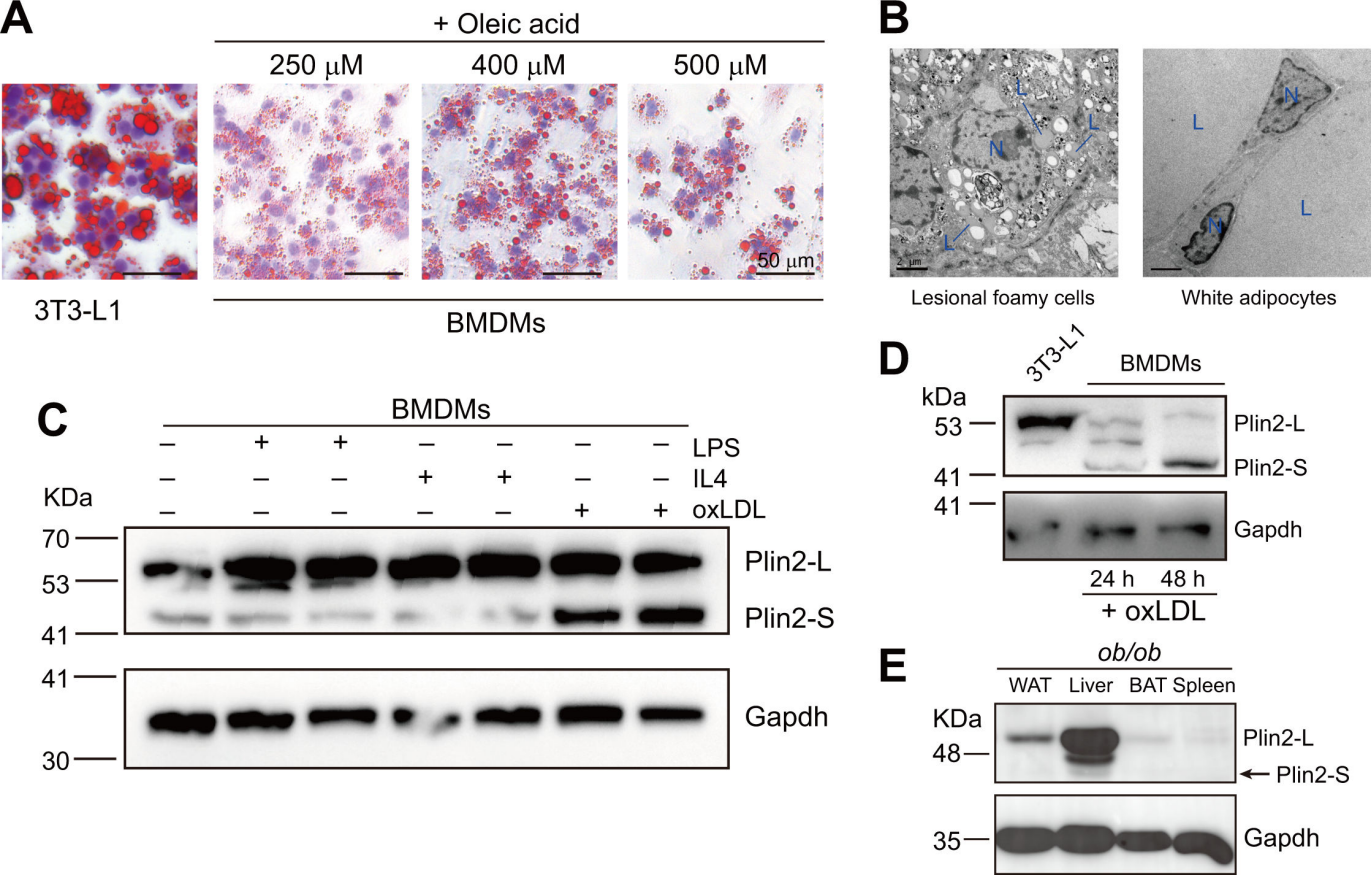
A truncated Plin2 isoform exists in foamy macrophages. **A.** Oil Red O staining in 3T3-L1 differentiated adipocytes and mouse bone marrow-derived macrophages (BMDMs) treated with 250 μM, 400 μM or 500 μM of oleic acid. **B**. Electron micrographs of lipid droplets in lesional cells of the atherosclerotic carotid artery and the white adipocytes of the adipose tissue. The carotid artery was isolated from an *Apoe^–/–^* mouse after 12 weeks of high-cholesterol diet feeding and the white adipose tissue was isolated from the obese *ob/ob* mouse. L: lipid droplet; N: nucleus. **C**. Western blots of Plin2 and Gapdh in BMDMs with or without the treatment of LPS, IL4, or oxidized LDL (oxLDL). Plin2-L indicates the full-length Plin2, while Plin2-S indicates the truncated isoform. **D**. Western blots of Plin2 and Gapdh in 3T3-L1 differentiated adipocytes and oxLDL-treated foamy macrophages. Plin2-L indicates the full-length Plin2, while Plin2-S indicates the truncated isoform. **E**. Western blots of Plin2 and Gapdh in white adipose tissue (WAT), liver, brown adipose tissue (BAT), and spleen isolated from obese *ob/ob* mice. Plin2-L indicates the full-length Plin2, while Plin2-S indicates the truncated isoform.

To figure out the potential underlying mechanism regulating heterogeneity of LDs between macrophages and adipocytes, we first investigated whether the LDs exhibited differences in size. Compared with adipocytes that were differentiated from 3T3-L1 fibroblasts, murine bone marrow-derived macrophages (BMDMs) displayed smaller LDs upon the treatment with oleic acid (OA; Figure 1A). We found that the maximum diameter of LDs in macrophages did not exceed 1.5 μm. When larger LDs approaching 1.5 μm in diameter appeared, macrophages exhibited significant intolerance, with markedly increased cell mortality (as OA concentration increased, LDs enlarged significantly, but adherent cells decreased substantially; Figure 1A). In contrast, 3T3-L1-differentiated adipocytes could tolerate LDs > 3 μm in diameter (Figure 1A). Similarly, the LD size in foamy cells from atherosclerotic plaques was significantly smaller than that in mouse white adipocytes, which had a unilocular LD (Figure 1B).

Considering that the PAT protein plays an important role in regulating the dynamic balance of lipid storage and degradation in LDs, and Plin2 is the primary member of the PAT family in macrophages, we examined Plin2 expression in lipid-laden foamy macrophages via Western blots. Of interest, we observed that LD induction by oxLDL or OA treatment dramatically promoted Plin2 truncation (Plin2-S), unlike LPS, IL-4 stimulation, or resting macrophages (Figure 1C and Supplementary Figure S1). However, in 3T3-L1 adipocytes, Plin2 was predominantly expressed as the full-length isoform (Plin2-L; Figure 1D). Consistent with this, the Plin2-L isoform was predominant in the white adipose tissue of obese *ob/ob* mice (Figure 1E). In the liver, however, a distinct isoform of intermediate size between Plin2-L and Plin2-S emerged as the second most abundant (Figure 1E).

The data demonstrate that Plin2 isoforms are produced in a cell- and tissue-specific manner, suggesting distinct functional roles across various cellular contexts.

### Plin2 protein could be truncated at the N-terminus

**Figure 2:**
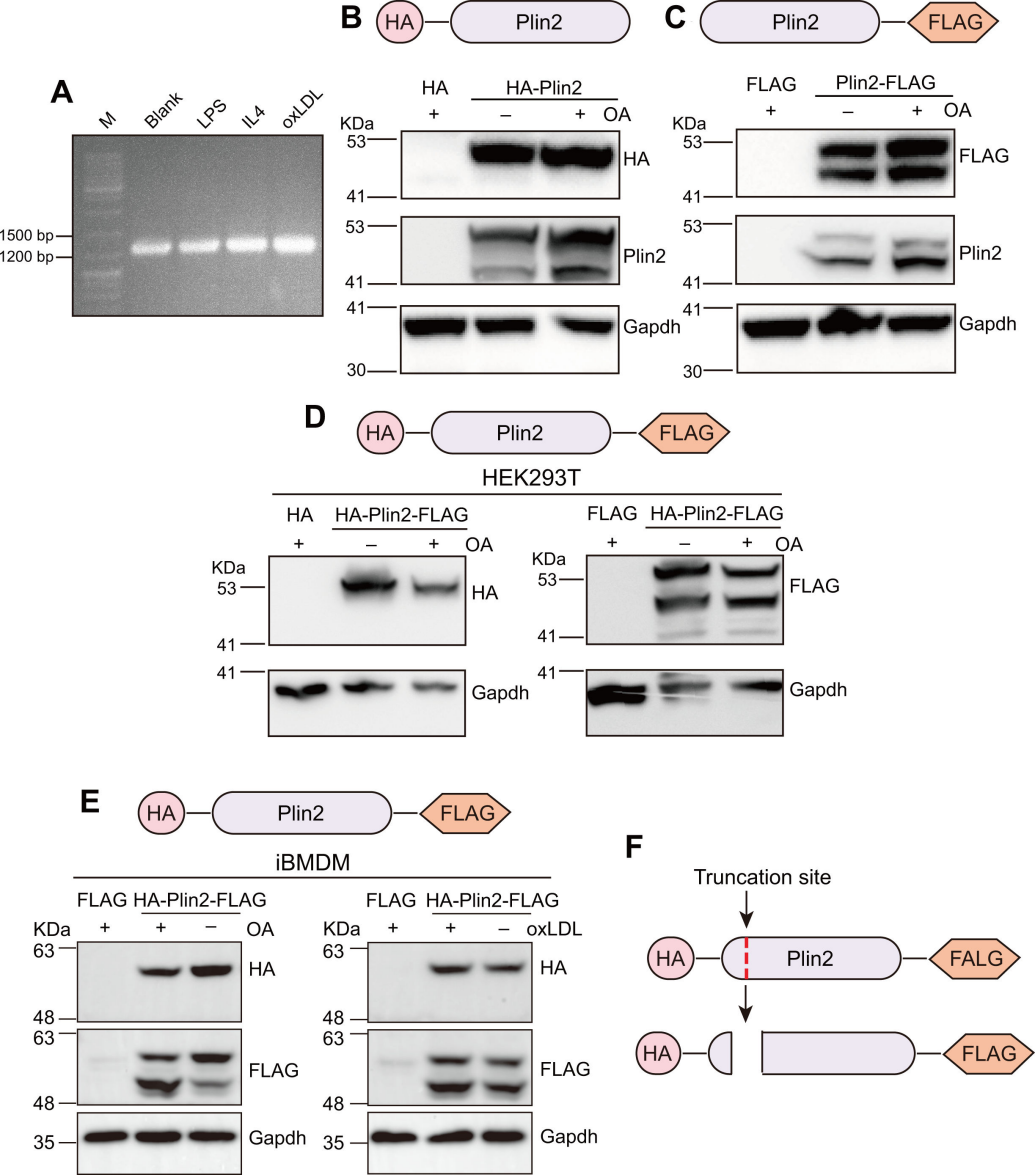
Plin2 could be truncated at its N-terminus. **A.** PCR amplification using primers targeting the 5' and 3' ends of the full-length *Plin2* coding sequence (CDS) from cDNA templates derived from BMDMs treated with or without LPS, IL-4, or oxLDL. PCR products were then resolved by agarose gel electrophoresis and visualized. M: DNA ladder. **B**. Western blots of HA, Plin2, and Gapdh in HEK293T cells transfected with HA plasmids fused with or without Plin2 at HA-tag’s C-terminus. Cells were treated with or without 500 µM oleic acid (OA) for 24 h. **C**. Western blots of FLAG, Plin2, and Gapdh in HEK293T cells transfected with FLAG plasmids fused with or without Plin2 at FLAG-tag’s N-terminus. Cells were treated with or without 500 µM OA for 24 h. **D and E**. Western blots of HA, FLAG, and Gapdh in OA-treated HEK293T cells (**D**), OA-treated iBMDMs (E, left), and oxLDL-treated iBMDMs (E, right). Cells were transfected with plasmids expressing Plin2 fused with HA-tag at its N-terminus and FLAG-tag at its C-terminus. HA or FLAG plasmids were used as controls. **F**. Schematic diagram of the truncation site of Plin2.

To determine whether the truncated Plin2 isoform arises from alternative splicing at the mRNA level or post-translational protein truncation, we first performed PCR amplifications using primers targeting the 5' and 3' ends of the full-length *Plin2* CDS with cDNAs from macrophages treated with or without LPS, IL-4, or oxLDL as templates. Agarose gel electrophoresis of the PCR products revealed a single band between 1,200 and 1,500 bp, corresponding to the full-length CDS of *Plin2* mRNA, regardless of treatment conditions (Figure 2A). Subsequent sequencing confirmed that the amplified product matched the 1,278 bp coding sequence of mouse *Plin2* published in the NCBI database. These results demonstrate that the alternative splicing does not account for the truncated Plin2 isoform observed in foamy macrophages.

Next, to elucidate the mechanism underlying Plin2 truncation in macrophages, we first mapped the truncation site to either the C- or N-terminus. To this aim, we generated recombinant plasmids encoding Plin2-L with the HA-tag fused to the N-terminus (HA-Plin2; Figure 2B) or with the FLAG-tag fused to the C-terminus (Plin2-FLAG; Figure 2C). Western blot analysis of OA-treated HEK293T cells transfected with these plasmids demonstrated that while HA-tagged Plin2 overexpression yielded two distinct bands detected by the anti-Plin2 antibody (against residues 350–425), the anti-HA antibody recognized only the higher molecular weight band (Figure 2B). However, similarly to the anti-Plin2 antibody, the anti-FLAG antibody detected both molecular weight Plin2 variants in HEK293T cells transfected with the Plin2-FLAG plasmid (Figure 2C).

To eliminate potential batch effects from using separate plasmids, we constructed a single Plin2 overexpression plasmid containing both tags, with an N-terminal HA tag and a C-terminal FLAG tag (Figure 2D). We then transfected this construct into HEK293T cells treated with OA or into immortalized BMDMs (iBMDMs) treated with OA or oxLDL. Consistent with results obtained from individual plasmids, the anti-FLAG antibody detected both molecular weight variants of Plin2, whereas the anti-HA antibody only recognized the higher molecular weight form in HEK293T cells (Figure 2D). Similar results were observed in iBMDMs (Figure 2E).

These results demonstrate that the N-terminal HA tag was cleaved in the lower molecular weight Plin2 variant, while the C-terminal FLAG tag remained intact in this form (Figure 2F). This indicates that Plin2 could be truncated at the N-terminus.

### The short Plin2 isoform is truncated at amino acids 40–44

**Figure 3:**
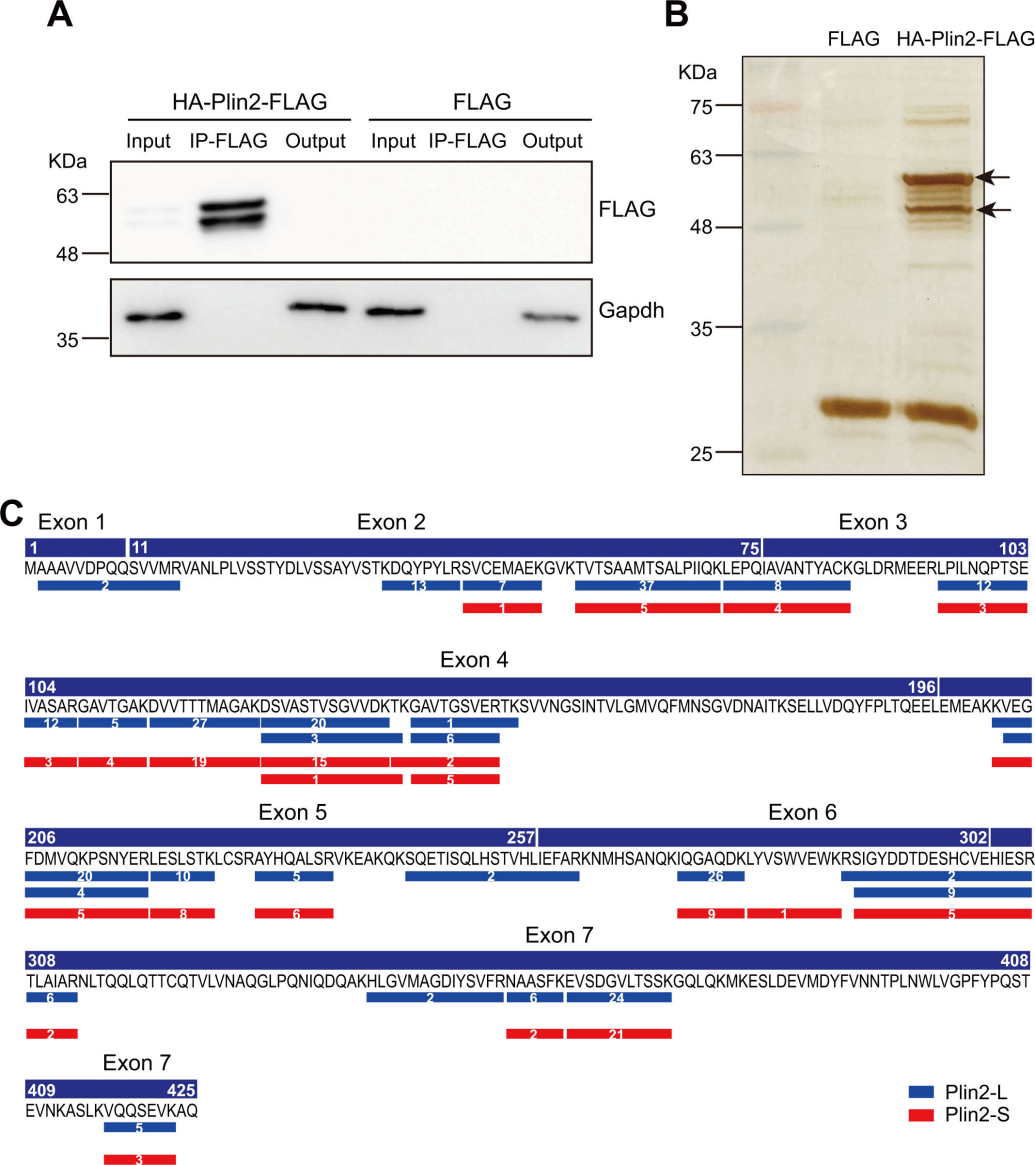
Plin2 was truncated among residues 40–44. **A.** Western blots of FLAG and Gapdh in HEK293T cells transfected with HA-Plin2-FLAG plasmids or FLAG control vectors. The FLAG antibody was used to purify FLAG-tagged Plin2 via Immunoprecipitation (IP). Cells were treated with oleic acid (500 µM) for 24 h. Input and output indicate cell lysates before and after IP. **B**. Silver staining of FLAG-immunoprecipitated proteins from HEK293T cells transfected with HA-Plin2-FLAG plasmids or FLAG control vectors. Cells were treated with oleic acid (500 µM) for 24 h. Bands that were cut for subsequent mass spectrometry analysis are indicated by arrows. **C.** Alignment of peptides obtained by the mass spectrometry analysis of bands shown in B with the NCBI-published mouse Plin2 protein sequence. Thin blue rectangles indicate peptides for the band with larger molecular weight (Plin2-L) and thin red rectangles indicate peptides for the band with smaller molecular weight (Plin2-S). Numbers in the rectangles indicate the peptide frequency among the mass spectrometry data.

To map the truncation site at the N-terminus of Plin2, we performed FLAG immunoprecipitation (IP) coupled with mass spectrometry analysis in OA-treated HEK293T cells transfected with HA-Plin2-FLAG. After verifying the IP efficiency by Western blot analysis (Figure 3A), we visualized the immunoprecipitated proteins on the polyacrylamide gel using silver staining, then excised gel bands corresponding to both full-length Plin2 and the truncated form (Figure 3B) for mass spectrometry analysis.

Using protein mass spectrometry data aligned with the NCBI-published mouse Plin2 protein sequence, we mapped peptide coverage for both full-length and truncated Plin2. The result revealed that residues 1–44 were absent in the truncated form (Figure 3C). Given the 5-amino acid detection threshold of mass spectrometry, we assumed that the cleavage occurs between residues 40 and 44 for the truncated isoform of Plin2.

### A noncanonical proteolytic pathway mediates Plin2 truncation

**Figure 4:**
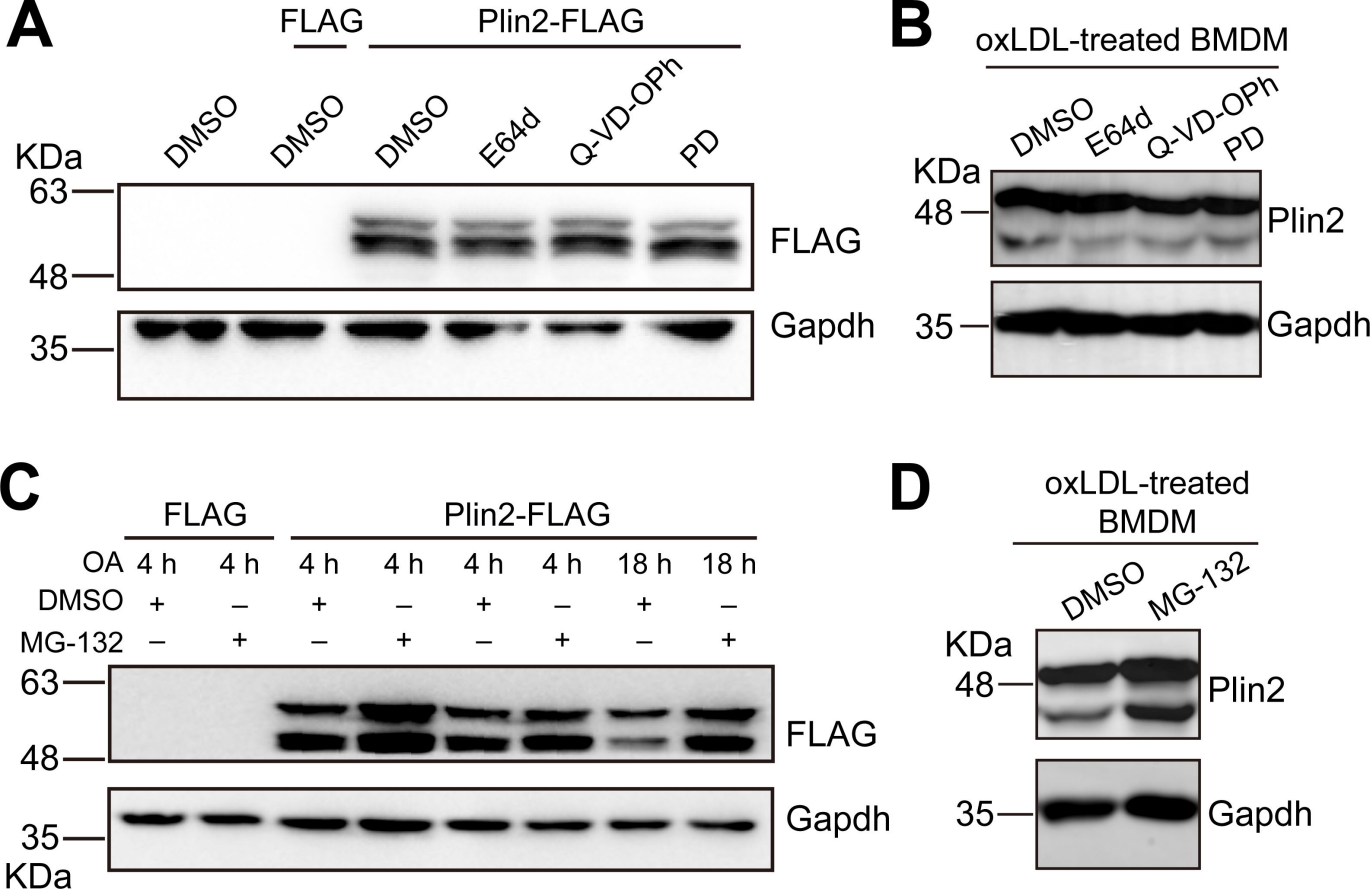
The classical protein hydrolysis pathways are not involved in the cleavage of Plin2 to generate the short isoform. **A.** Western blots of FLAG and Gapdh in HEK293T cells transfected with Plin2-FLAG plasmids or FLAG control vectors and treated with or without protease inhibitors E64D, Q-VD-OPh, and PD as indicated. Lipid droplets were induced by treating cells with oleic acid. **B.** Western blots of Plin2 and Gapdh in BMDMs treated with or without protease inhibitors E64D, Q-VD-OPh, and PD as indicated. Lipid droplets were induced by treating cells with oxLDL. **C**. Western blots of FLAG and Gapdh in HEK293T cells transfected with Plin2-FLAG plasmids or FLAG control vectors and treated with or without the proteasome inhibitor MG-132 as indicated. Lipid droplets were induced by treating cells with oleic acid. **D.** Western blots of Plin2 and Gapdh in BMDMs treated with or without the proteasome inhibitor MG-132. Lipid droplets were induced by treating cells with oxLDL.

To determine the potential mechanism underlying Plin2 truncation, we first treated OA-loaded HEK293T cells overexpressing Plin2-FLAG with several broad-spectrum protease inhibitors, including Aloxistatin (E64D, a cysteine protease inhibitor), Q-VD-OPh (a pan-caspase inhibitor), and PD (a calpain inhibitor). The inhibitory efficacies of these inhibitors against their respective target proteases were first verified by measuring the activities of Cathepsin B (for E64D), Caspase-1 (for Q-VD-OPh), and Calpain (for PD) (Supplementary Figure S2A-C), respectively. Subsequent Western blot analysis revealed that these inhibitors did not prevent Plin2 truncation (Figure 4A), suggesting that proteolytic enzymes likely do not mediate the cleavage. This finding was further confirmed in oxLDL-treated macrophages (Figure 4B).

Next, we examined whether the ubiquitin-proteasome system mediates Plin2 truncation by treating the cells with the proteasome inhibitor MG-132. Treatment of cells with MG-132 led to a pronounced accumulation of high-molecular-weight ubiquitinated proteins, as detected by Western blot analysis using an anti-ubiquitin antibody (Supplementary Figure S2D). However, MG-132 failed to block truncation of Plin2 in either HEK293T cells (Figure 4C) or oxLDL-treated macrophages (Figure 4D), indicating that this process is independent of ubiquitin-proteasome degradation.

Together, these findings suggest that Plin2 is processed through a nonclassical proteolytic pathway.

### Truncated Plin2 causes smaller LD generation

**Figure 5:**
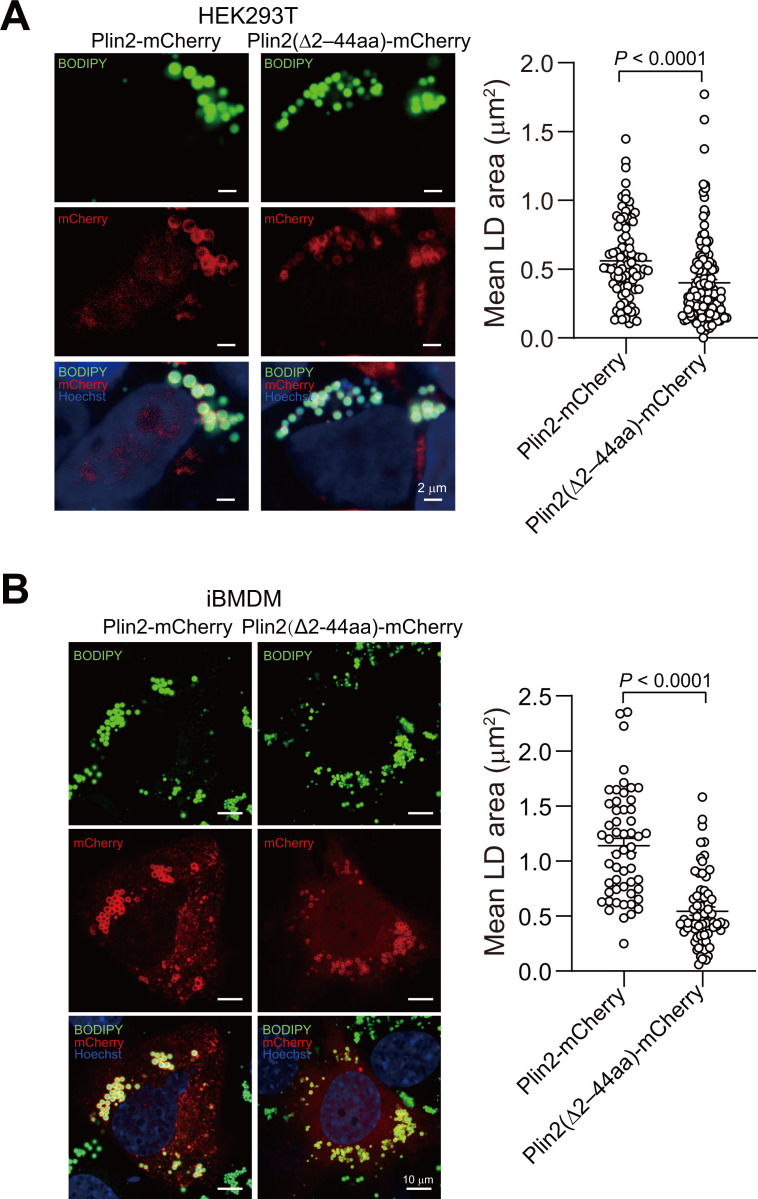
N-terminally truncated Plin2 limits LD size. **A and B.** Confocal imaging and size analysis of lipid droplets in HEK293T cells (**A**) and iBMDMs (**B**) transfected with recombinant plasmids expressing Plin2-mCherry fusion proteins, either containing or lacking (Δ2–44aa) the 2–44 residues. Lipid droplets were induced by treating cells with oleic acid for 24 h (**A**) or with oxLDL for 48 h (**B**) and stained with BODIPY. Total cell counts for Plin2-mCherry and Plin2(Δ2–44aa)-mCherry groups were *n* = 91/140 (HEK293T) and *n* = 54/71 (iBMDMs). The data are shown as means ± SEMs and 2-tailed unpaired Student’s *t*-tests were performed.

To examine the role of the truncated Plin2 protein (Plin2-S) in LD regulation, we generated a recombinant plasmid encoding the Plin2 protein variant with a 2–44 amino acid deletion (Δ2–44aa) fused to mCherry and overexpressed it in HEK293T cells. Following treatment with OA, the confocal microscopic analysis revealed that cells expressing Plin2(Δ2–44aa) exhibited significantly smaller individual LDs compared with those expressing full-length Plin2 (Figure 5A). A similar phenotype was also observed in iBMDMs treated with oxLDL (Figure 5B). These findings suggest that Plin2 truncation at the N-terminus may modulate LD heterogeneity, specifically influencing size distribution across cell types.

## Discussion

Metabolic disorders such as obesity, fatty liver disease, and atherosclerosis are closely associated with intracellular LD load capacity [15–18]. Notably, the lipid-storage capacity of LDs exhibits remarkable heterogeneity across cell types. For instance, adipocytes can form exceptionally large unilocular LDs, whereas hepatocytes and macrophages typically contain smaller LDs with comparatively reduced lipid-storage capacity. Deciphering the molecular basis of LD heterogeneity may therefore provide crucial insights into the pathogenesis of metabolic diseases and potentially reveal novel therapeutic targets. In this study, we identified an N-terminally truncated form of Plin2, which was present in foamy macrophages but absent in adipocytes. MS analysis indicated the deletion of the first 44 amino acids in this truncated isoform, and forced expression of this variant reduced LD size. These findings suggest that macrophages regulate LD size by expressing specific Plin2 variants, potentially offering a mechanism to enhance lipid-storage capacity in these cells.

Consistent with our finding, an N-terminal-specific anti-Plin2 antibody detected only a single ~ 50 kDa band, while a C-terminal-specific antibody, as that we used in this study, identified both this band and an additional ~ 43 kDa truncated isoform in mouse mammary tissue lysates [19]. Functional characterization demonstrated that although this truncated Plin2 isoform retained the ability to mediate LD formation, its efficiency was significantly reduced compared with full-length Plin2. These findings suggest the potential existence of naturally occurring truncated Plin2 isoforms in vivo that maintain the core functionality of Plin2 as an LD-associated protein but with compromised lipid-storage capacity.

The N-terminus of Plin2 contains a highly conserved PAT domain, yet this domain neither binds cholesterol/stearic acid nor mediates Plin2’s localization to the phospholipid monolayer of LDs [20,21]. However, studies demonstrate that the N-terminal sequence of Plin2 is critical for ubiquitin-proteasome-mediated degradation of cytosolic Plin2 [14]. Notably, human PLIN2 lacking the first 89 amino acids remains stable and escapes degradation even when unbound to LDs. Furthermore, the N-terminus of Plin2 obstructs another PAT protein, Plin3, from LD binding—down-regulation of Plin2 in macrophages promotes Plin3 relocation from the plasma membrane to LDs [13]. These findings suggest that the PAT domain dynamically regulates Plin2 protein stability and modulates LD protein composition in response to cellular lipid metabolic states. We demonstrated that deletion of the N-terminal 44 amino acids in Plin2 reduces LD size, indicating a critical functional role for the highly conserved PAT domain in LD biology—a finding warranting further investigation.

Moreover, while we inhibited several canonical protein degradation pathways to investigate the mechanism underlying Plin2 truncation, none of these interventions prevented truncation. This suggests the involvement of either a noncanonical mechanism or compensatory pathway. Our LC-MS/MS analysis of the purified Plin2-S isoform pinpointed the 40–44 (YPYLR) motif as the site of truncation. The presence of proline (P41) within this motif is highly significant; while proline typically creates a structural barrier for common proteases, specialized enzymes such as prolyl oligopeptidases (POP) specifically cleave the carboxyl side of proline residues [22]. The unique size-exclusion gating of such enzymes allows them to target flexible, disordered segments like the Plin2 N-terminal tail while leaving the globular core of the protein intact. Together, our data suggest that the 40–44 region serves as a regulated processing site for specialized peptidases that need further investigation.

In summary, our study identified a specific truncated isoform of Plin2 that promotes the formation of smaller LDs in macrophages. This finding suggests a potential strategy to enhance macrophage lipid-storage capacity, which could mitigate lipid overload-induced cell death in metabolic disorders such as atherosclerosis.

## Materials and methods

### Animal models

Fifteen-week-old male B6.Cg-*Lep^ob^*/J (*ob/ob*) mice were purchased from GemPharmatech for tissue harvest. *Apoe^–/–^* mice were fed with the high-cholesterol diet (Research diet; #D12079B) for 12 weeks for the transmission electron microscopy experiment as described below. C57BL/6 mice were used for bone marrow isolation. Mice were anesthetized with inhalation of 3.5% isoflurane in an induction box and then were attached to an anesthetic mask for maintenance with 1.5% isoflurane. Mice were killed by cervical dislocation. All mice were maintained in a specific-pathogen-free barrier facility.

### Cell culture and stimulation

Bone marrow cells were isolated and harvested from the tibias and femurs of killed male or female mice and cultured in DMEM/F12 + GlutaMAX medium (Thermo; #10565–018) supplemented with 15% L929 conditioned medium, 10% FBS (Gibco; #10099–141C), and 1‰ gentamicin (Sigma; #345815) at 37°C with 5% CO2. Half of the medium was replaced with fresh medium on days 3 and 5. If applicable, the BMDMs were treated with oxidized low-density lipoprotein (oxLDL, 100 μg/ml; YEASEN; #20605ES05) for 48 h or oleic acid (OA, 250 μM; Sigma; #O1008-1G) for 24 h to induce LDs after seven days of culture. Macrophages were stimulated with lipopolysaccharide (LPS, 100 ng/ml; Sigma; #L5293) or IL4 (5 ng/ml; Peprotech; #214–14-20) for 24 h in some experiments. Additionally, iBMDMs were cultured in DMEM/F12 + GlutaMAX medium supplemented with 10% FBS and 1‰ gentamicin at 37°C with 5% CO2. LD induction in iBMDMs was performed as described for primary BMDMs.

HEK293T cells were cultured in DMEM medium supplemented with 10% FBS and 1‰ gentamicin at 37°C with 5% CO2. To induce LDs, HEK293T cells were treated with 500 μM OA for 24 h.

To investigate the mechanism of Plin2 processing, HEK293T cells were treated with the protease inhibitors Aloxistatin (E64D, 20 μM; MCE; #HY-12305), Q-VD-OPh (20 μM; MCE; #HY-100229), or PD151746 (PD, 20 μM; MCE; #HY-19749) for 24 h or treated with the proteasome inhibitor MG-132 (10 μM; MCE; #HY-13259) for 4 h, in the presence of OA treatment for either 4 or 18 h. To assess the inhibitory efficiency of E64D, cells were lysed in a buffer comprising 50 mM Tris-HCl (pH 8.0), 150 mM NaCl, 1% NP-40, 0.25% sodium deoxycholate, and 1 mM EDTA. Lysates were incubated for 35 min in an assay buffer (50 mM sodium acetate, pH 5.5, 0.1 M NaCl, 1 mM EDTA, and 0.2% Triton X-100) supplemented with 20 μM of the cathepsin B substrate Z-Arg-Arg-AMC hydrochloride (MCE; #HY-134434). Fluorescence was then measured using a Multi-detection microplate reader (Infinite M200 Pro, TECAN) at excitation and emission wavelengths of 380 nm and 460 nm, respectively. Additionally, the inhibitory efficiencies of Q-VD-OPh and PD were evaluated using a Caspase-1 Activity Assay Kit (MCE; #HY-K2610) and a Calpain Activity Assay Kit (Beyotime; #P0375S), respectively. Finally, the efficacy of MG-132 was determined by Western blot analysis of ubiquitinated protein levels using an anti-ubiquitin antibody (MCE; #HY-P80925). These inhibitors were applied to oxLDL-loaded BMDMs at the same concentrations and for the same durations as used in HEK293T cells.

3T3-L1 cells were cultured in DMEM medium supplemented with 10% NCS (Gibco; #16010159) and 1‰ gentamicin at 37°C with 5% CO2. To induce 3T3-L1 cells to differentiate to adipocytes, cells were cultured in DMEM medium supplemented with 10% FBS (Gibco; #10099–141) and 1‰ gentamicin in the presence of pro-differentiative agents, including insulin (1 μg/ml; Sigma; #I6634), dexamethasone (1 μM; Sigma; #D4902), IBMX (0.5 mM; Sigma; #I7018) and rosiglitazone (2 μM; Sigma; #R2408), for seven days.

### Confocal imaging for LDs

HEK293T cells were seeded at 4 × 10^5^ cells/ml and iBMDMs were seeded at 2 × 10^5^ cells/ml in confocal dishes (Cellvis; #D35C4-20-1-N). The cells were transfected with recombinant plasmids for 48 h and treated with 500 μM OA for 24 h (for HEK293T) or with oxLDL for 48 h (for iBMDMs), followed by fixation in 4% paraformaldehyde for 15 min. After washing with PBS twice, cells were incubated with 2 μM BODIPY (Thermo; #D3922) and 2 μg/ml Hoechst (YEASEN; #33342) at 37°C for 25 min. Cells were rinsed in DMEM medium after washing with pre-warmed PBS twice and then visualized using a confocal microscope (Nikon; AX R with NSPARC).

### Immunoprecipitation (IP) and mass spectrometry

Cells were scraped and lysed in the IP lysis buffer (Thermo; #87787) supplemented with freshly added protease inhibitor cocktail. After centrifugation at 4°C for 15 min, cleared lysates were collected and protein concentrations were determined using a BCA Protein Quantification Kit (YEASEN; #20201ES76). HA antibody-conjugated magnetic beads were prepared by incubating Protein A/G magnetic beads (Thermo; #26162) with anti-HA antibody (ABclonal; #AE008). For immunoprecipitation, lysates were incubated with either anti-FLAG Magnetic Beads (Thermo; #A36797) or HA antibody-conjugated magnetic beads at 4°C for 25 min with rotation. Beads were collected using a Magna GrIPTM Rack (Millipore; #20–400) and washed three times with PBS. Bound proteins were eluted with glycine buffer (0.1 M, pH 2.8) and immediately neutralized with Tris buffer (1 M, pH 8.5). Eluates were analyzed by Western blot or subjected to silver staining with a Fast Silver Stain Kit (Beyotime; #P0017S) followed by mass spectrometry (MS) identification.

After IP, bands of interest were excised from the silver-stained PAGE gel and subjected to mass spectrometry (MS) analysis. The gel bands were digested with trypsin, and the resulting peptides were analyzed using an LC-MS/MS system (ThermoFisher; Easy-nLC 1000 & Q Exactive). Peptide identification was performed using the MASCOT mass spectrometry matching software to obtain qualitative data on the target protein peptide molecules (Supplementary Table S1 and Supplementary Table S2).

### Transmission electron microscopy imaging

The carotid artery was isolated from an *Apoe^–/–^* mouse after 12 weeks of high-cholesterol diet (Research diet; #D12079B) feeding and the white adipose tissue (WAT) was isolated from the obese *ob/ob* mouse followed by fixation in 2.5% glutaraldehyde overnight. After washing twice with 100  mM phosphate buffer, the samples were fixed with 1% osmium tetroxide for 1.5 h, and then dehydrated in a step-up graded series of ethanol, cleared in acetone, and infiltrated in fresh 100% resin successively. Finally, the samples were polymerized at 60°C for 48  h and embedded in Epon 812 resin followed by sectioning. Images were acquired using a transmission electron microscope (FEI Tecnai G2 Spirit).

### Western blot

Cells or tissues were lysed in RIPA buffer supplemented with freshly added protease inhibitor cocktail. Proteins were resolved on 10% or 12% SDS–PAGE gels and were transferred to PVDF membranes. After blocking in 5% milk/TBST, the membranes were incubated with primary antibodies diluted in 5% milk/TBST overnight at 4°C. After washing in TBST three times, HRP-conjugated secondary antibodies were added and incubated for 1 h at room temperature. After washing, signals were developed with SuperSignal West Pico PLUS ECL substrates (Thermo; #34580). The antibodies used for Western blots were listed as below: anti-Plin2 (1:625; Novus Biological; #NB 110–40877SS; against residues 350–425), anti-FLAG (1:2,000; CWBIO; #CW0287M), anti-HA (1:2,000; CWBIO; #CW0092M), anti-ubiquitin (1:1,000; MCE; #HY-P80925), anti-Gapdh (1:20,000; Proteintech; #60004–1-Ig), anti-rabbit IgG (1:5,000; Jackson Immuno; #111–035-144), and anti-mouse IgG (1:5,000; Jackson Immuno; #115–035-003).

### Oil-red-O staining

3T3-L1 differentiated adipocytes and OA-treated BMDMs were fixed in 4% paraformaldehyde for 15 min. After washing with PBS twice, cells were rinsed quickly in 60% isopropanol for 15 s followed by complete drying at room temperature (RT). Cells were then stained with 60% Oil Red O solution (Sigma; #O0625-25G) at RT for 10 min. After removing Oil Red O solution, cells were immediately rinsed in 60% isopropanol for 15 s followed by washing with PBS for three times. Cells were counterstained with hematoxylin (Beyotime; #C0107) for imaging with a bright-field microscope (DM4B, Leica) connected to a CCD camera.

### RNA isolation, reverse transcription, and polymerase chain reactions (PCRs)

Total RNA was isolated using the MiPure Cell/Tissue miRNA Kit (Vazyme; #RC201) and reverse-transcribed into cDNA with the GoScript™ Reverse Transcription Mix (Promega; #A2801). To detect *Plin2* mRNA variants, PCR amplification was performed using primers (Forward: 5'-ATGGCAGCAGCAGTAGTG, Reverse: 5'-TTACTGAGCTTTGACCTCAGAC) targeting the 5' and 3' ends of the full-length *Plin2* coding sequence (CDS). The cDNA templates were derived from BMDMs treated with or without LPS, IL-4, or oxLDL, and reactions were carried out using the TransStart FastPfu PCR SuperMix (TransGen Biotech; #AS221-01). PCR products were then resolved by agarose gel electrophoresis and visualized with a Tanon MINI Space1000 gel imaging system.

### Plasmid construction and transfection

The pcDNA3.1-HA-N (Fenghui Bio; #ZT119) and pcDNA3.1–3xFLAG-C (Fenghui Bio; #BR084) plasmids linearized with XhoI and KpnI were used to construct HA-Plin2 and Plin2-FLAG recombinant plasmids, respectively. The pcDNA3.1-HA-N plasmids linearized with XhoI and KpnI were also used to construct HA-Plin2-FLAG recombinant plasmids. The pmCherry-N1 (Fenghui Bio; #BR049) vectors linearized with XhoI and BamHI were used to construct Plin2-mCherry recombinant plasmids. Insert gene fragments were amplified by PCRs using primers (Supplementary Table S3) with cohesive ends corresponding to the restriction enzymes and purified using the EasyPure PCR Purification Kit (TransGen Biotech; #EP101-01). Subsequently, the linearized backbone and the insert gene fragments were ligated with the T4 ligase (TransGen Biotech; #FL101-02). The ligation products were transformed into Trans10 Chemically Competent Cells (TransGen Biotech; #CD101-01). The recombinant plasmids were isolated with a Plasmid Mini Kit (Qiagen; #12123), which were confirmed that the inserted gene is correct through sequencing. The recombinant plasmids with deletion of residues 2–44 [Plin2(Δ2–44aa)-mCherry] were constructed using the Plin2-mCherry plasmid as a template through a homologous recombination method (primer sequences are listed in Supplementary Table S3).

HEK293T cells were transfected with the recombinant plasmids using Lipofectamine 3000 (Thermo; #L3000001) at the concentration of 1 μg/ml. iBMDMs were transfected with the recombinant plasmids using Lipofectamine LTX (Thermo; #15338100) at the concentration of 1.25 μg/ml. After 48 h, cells were collected for Western blot, IP, or visualization by a confocal microscopy.

## Supplementary material

Online supplementary figure 1

## Data Availability

All data supporting the findings of this study are available from the corresponding author upon reasonable request. All data generated or analyzed during this study are included in this published article (and its Supplemental Materials).

## Abbreviations

BMDMs: bone marrow-derived macrophages
CDS: coding sequence
ER: endoplasmic reticulum
IP: immunoprecipitation
LDs: lipid droplets
OA: oleic acid
POP: prolyl oligopeptidases
iBMDMs: immortalized bone marrow-derived macrophages
oxLDL: oxidized low-density lipoprotein
