# Supplementary material for: Macrophage-specific N-terminal truncation of Plin2 limits the size of lipid droplets
Source: Biochem J. 2026 Jan 21;483(2):107–18. doi: 10.1042/BCJ20253345 (PMC12921128; doi:10.1042/BCJ20253345)
Supplement: Online supplementary figure 1 [file bcj-483-2-BCJ20253345-s001.pdf]

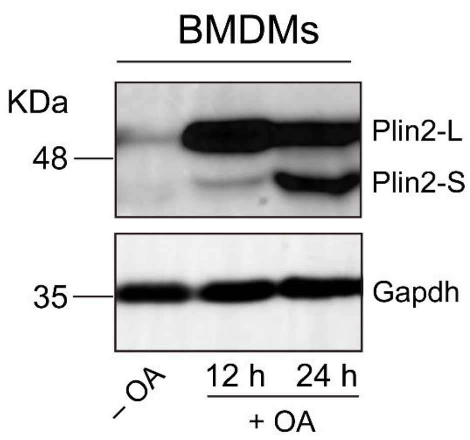

Figure S1. A truncated Plin2 isoform exists in OA-treated macrophages. Western blots of Plin2 and Gapdh in BMDMs with or without the treatment of OA. Plin2-L indicates the full-length Plin2, while Plin2-S indicates the truncated isoform.

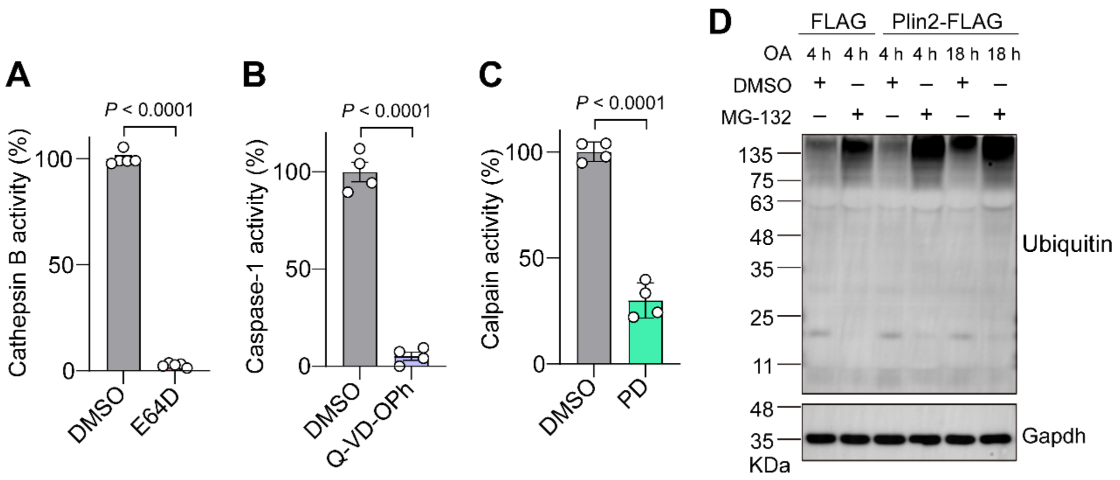

Figure S2. Validation of protease and proteasome inhibitors in HEK293T cells.

A. The inhibitory efficacy of E64D was confirmed by measuring the activity of cathepsin B using the fluorogenic substrate Z-Arg-Arg-AMC. n = 5.

B. The efficacy of the pan-caspase inhibitor Q-VD-OPh was evaluated via Caspase-1 activity using the chromogenic substrate Ac-YVAD-pNA. n = 4.

C. Calpain activity was assessed using a fluorogenic substrate that releases 7-amino-4-trifluoromethylcoumarin (AFC) upon cleavage. n = 4.

D. Western blot analysis using an anti-ubiquitin antibody confirmed the efficacy of MG-132. MG-132-treated cells displayed an intense smear of high-molecular-weight ubiquitinated proteins (starting above 75 kDa), indicating the stabilization of polyubiquitinated proteins due to 26S proteasome inhibition.

Table S1. Amino acid sequence of full-length Plin2 protein peptides identified by MS.

Annotated Sequence	# PSMs	Master Protein Accessions	Positions in Master Proteins
[R].LPILNQPTSEIVASAR.[G]	12	P43883	P43883 [94-109]
[K].IQGAQDK.[L]	26	P43883	P43883 [272-278]
[R].NAASFK.[E]	6	P43883	P43883 [357-362]
[K].EVSDGVLTSSK.[G]	24	P43883	P43883 [363-373]
[K].HLGVMAGDIYSVFR.[N]	2	P43883	P43883 [343-356]
[K].LEPQIAVANTYACK.[G]	8	P43883	P43883 [72-85]
[R].LESLSTK.[L]	10	P43883	P43883 [218-224]
[K].GAVTGSVER.[T]	6	P43883	P43883 [143-151]
[K].KVEGFDMVQKPSNYER.[L]	8	P43883	P43883 [202-217]
[K].DVVTTTMAGAK.[D]	9	P43883	P43883 [117-127]
[K].DVVTTTMAGAK.[D]	18	P43883	P43883 [117-127]
[K].TVTSAAMTSALPIIQK.[L]	9	P43883	P43883 [56-71]
[K].TVTSAAMTSALPIIQK.[L]	28	P43883	P43883 [56-71]
[R].SIGYDDTDESHCVEHIESR.[T]	9	P43883	P43883 [289-307]
[K].DSVASTVSGVVDKTK.[G]	3	P43883	P43883 [128-142]
[K].DSVASTVSGVVDK.[T]	20	P43883	P43883 [128-140]
[K].SQETISQLHSTVHLIEFAR.[K]	2	P43883	P43883 [244-262]
[R].TLAIAR.[N]	6	P43883	P43883 [308-313]
[R].SVCEMAEK.[G]	4	P43883	P43883 [45-52]
[K].KVEGFDMVQKPSNYER.[L]	12	P43883	P43883 [202-217]
[M].AAAVVDPQQSVVMR.[V]	2	P43883	P43883 [2-15]
[R].GAVTGAK.[D]	5	P43883	P43883 [110-116]
[R].AYHQALSR.[V]	5	P43883	P43883 [229-236]
[K].VQQSEVK.[A]	5	P43883	P43883 [417-423]
[K].RSIGYDDTDESHCVEHIESR.[T]	2	P43883	P43883 [288-307]
[K].GAVTGSVERTK.[S]	1	P43883	P43883 [143-153]
[K].VEGFDMVQKPSNYER.[L]	4	P43883	P43883 [203-217]
[K].DQYPYLR.[S]	13	P43883	P43883 [38-44]
[R].SVCEMAEK.[G]	3	P43883	P43883 [45-52]

Note:

Accessions: Protein ID in the protein sequence database (FASTA database).

PSMs (peptide spectrum matches): Total number of mass spectra matched to peptides.

P43883: Mouse Plin2 protein ID.

Table S2. Amino acid sequence of truncated Plin2 protein peptides identified by MS.

Annotated Sequence	# PSMs	Master Protein Accessions	Positions in Master Proteins
[K].LEPQIAVANTYACK.[G]	4	P43883	P43883 [72-85]
[K].GAVTGSVER.[T]	5	P43883	P43883 [143-151]
[R].SIGYDDTDESHCVEHIESR.[T]	5	P43883	P43883 [289-307]
[R].TLAIAR.[N]	2	P43883	P43883 [308-313]
[K].KVEGFDMVQKPSNYER.[L]	3	P43883	P43883 [202-217]
[R].LESLSTK.[L]	8	P43883	P43883 [218-224]
[R].LPILNQPTSEIVASAR.[G]	3	P43883	P43883 [94-109]
[K].IQGAQDK.[L]	9	P43883	P43883 [272-278]
[K].LYVSWVEWK.[R]	1	P43883	P43883 [279-287]
[K].DVVTTTMAGAK.[D]	5	P43883	P43883 [117-127]
[K].EVSDGVLTSSK.[G]	21	P43883	P43883 [363-373]
[K].TVTSAAMTSALPIIQK.[L]	6	P43883	P43883 [56-71]
[K].TVTSAAMTSALPIIQK.[L]	4	P43883	P43883 [56-71]
[K].DSVASTVSGVVDK.[T]	15	P43883	P43883 [128-140]
[R].AYHQALSR.[V]	6	P43883	P43883 [229-236]
[K].DVVTTTMAGAK.[D]	14	P43883	P43883 [117-127]
[K].VQQSEVK.[A]	3	P43883	P43883 [417-423]
[R].SVCEMAEK.[G]	1	P43883	P43883 [45-52]
[R].NAASFK.[E]	2	P43883	P43883 [357-362]
[K].KVEGFDMVQKPSNYER.[L]	2	P43883	P43883 [202-217]
[R].GAVTGAK.[D]	4	P43883	P43883 [110-116]
[K].TKGAVTGSVER.[T]	2	P43883	P43883 [141-151]
[K].DSVASTVSGVVDKTK.[G]	1	P43883	P43883 [128-142]

Note:

Accessions: Protein ID in the protein sequence database (FASTA database).

PSMs (peptide spectrum matches): Total number of mass spectra matched to peptides.

P43883: Mouse Plin2 protein ID.

Table S3. The primer sequences used for plasmid construction.

Plasmid name	Sequences（5′→ 3′）	
HA-Plin2	F1: CATGGCAGCAGCAGTAGTG	F2: GTACCATGGCAGCAGCAGTAGTG
	R1: GTTACTGAGCTTTGACCTCAGAC	R2: TCGAGTTACTGAGCTTTGACCTCAGAC
Plin2-Flag	F1: CATGGCAGCAGCAGTAGTG	F2: GTACCATGGCAGCAGCAGTAGTG
	R1: GCTGAGCTTTGACCTCAGACTG	R2: TCGAGCTGAGCTTTGACCTCAGACTG
HA-Plin2-Flag	F1: CATGGCAGCAGCAGTAGTG	F2: GTACCCATGGCAGCAGCAGTAGTG
	R1: GCTTGTCATCGTCATCCTTGTAG	R2: TCGAGCTTGTCATCGTCATCCTTGTAG
Plin2-Cherry	F1: G ATGGCAGCAGCAGTAGTGG	F2: TCGAGATGGCAGCAGCAGTAGTGG
	R1: GATC CTGAGCTTTGACCTCAGAC	R2: CTGAGCTTTGACCTCAGACTGC
Plin2(Δ2–44aa)-Cherry	F: TCTCGAGATGTCCGTGTGTGAGATGGCCGA	
	R: CACACACGGACATCTCGAGATCTGAGTCCG	
